# Clip-Based Endoscopic Anastomotic Reduction in Patients with Dumping Syndrome and Postbariatric Hypoglycemia after Roux-en-Y Gastric Bypass

**DOI:** 10.1055/a-2931-7366

**Published:** 2026-08-20

**Authors:** Armin Kuellmer, Matthias Kandler, Sandra Nagl, Katharina Laubner, Maximilian Meyer-Steenbuck, Julius Mueller, Rafael Kaeser, Lukas Sturm, Jochen Seufert, Robert Thimme, Arthur Schmidt

**Affiliations:** 1 Department of Medicine II, Medical Center 88751 University of Freiburg Faculty of MedicineFreiburgBWGermany; 2 Department of Gastroenterology and EndoscopyDresden City HospitalDresdenSNGermany; 3 Department of Gastroenterology 39694 University Hospital AugsburgAugsburgBYGermany; 4 Division of Endocrinology and Diabetology, Department of Medicine II, Medical Center 88751 University of Freiburg Faculty of MedicineFreiburgBWGermany; 5 Berta-Ottenstein-Programme, Faculty of Medicine 88751 University of FreiburgFreiburgBWGermany; 6 Department of Gastroenterology, Hepatology, Endocrinology 15000 Bosch Health Campus, Robert Bosch HospitalStuttgartBWGermany

**Keywords:** GI surgery, endoscopy upper GI tract, motility/achalasia

## Abstract

**Background and Aims** Dumping syndrome (DS) and postbariatric hypoglycemia (PBH) represent common complications after Roux-en-Y gastric bypass (RYGB). Transoral outlet reduction (TORe) addresses the rapid passage of food through the gastrojejunal anastomosis which is central in pathophysiology. In this prospective multicenter study, technical feasibility and short-term clinical efficacy of a new clip-based endoscopic system (BARS) were evaluated.

**Methods** Twenty-three consecutive patients at three centers in Germany were included. The primary endpoint was feasibility measured as reduction of anastomotic diameter and surface. Secondary endpoints included changes of the Dumping symptom score and glycaemia in modified oral glucose tolerance tests (mod. oGTT), anastomotic diameter, quality of life after 12 weeks and adverse events.

**Results** The procedure was technically feasable in all cases. Median anastomotic diameter was reduced from 40 mm (IQR: 30–50) to 9 mm (IQR: 7–10); −77%) at baseline ( *p* < 0.001). At week 12, median Sigstad-Score decreased from 17,5 (IQR: 12.8–21.0) to 5 (IQR: 0–11; *p* < 0.001). Median anastomotic diameter was still reduced to 20 mm (IQR: 12–30; *p* = 0.004; −47%). Modified oGTT demonstrated abolished DS in 3 of 23 patients (13%) while no effect was seen in PBH at 12 weeks. Median BMI decreased from 30.6 kg/m ^2^ (IQR: 28–35.6) to 29.25 kg/m ^2^ (IQR: 26.7–33.4; *p* = 0.001). Quality of life (QoL) improved significantly in five of eight subcategories of the SF-36.

**Conclusion** Clip-based anastomotic reduction for DS and PBH seems feasible, safe and showed significant improvements in Sigstad scores and QoL in short-term analysis.

## Introduction

Dumping syndrome (DS) and postbariatric hypoglycemia (PBH) a represent common complications after Roux-en-Y bypass (RYGB). 1 2 The rapid passage of hyperosmolar food from the gastric pouch over the (enlarged) gastrojejunal anastomosis into the upper jejunum plays a crucial pathophysiologic role. This creates a fluid shift into the lumen and triggers release of several gastrointestinal hormones, including vasoactive agents, incretins, and glucose modulators. DS, formerly referred to as “early” dumping, occurs within the first hour after a meal and includes gastrointestinal and vasomotor symptoms. PBH, which was previously subsumed under “late” dumping, is at least partly due to the incretin effect with a postprandial increase in glucagon like peptide 1 (GLP-1) after RYGB. 1

Endoscopic treatment options aiming to reduce the diameter of the anastomosis are referred to as transoral endoscopic outlet reduction (TORe). The mechanism of action is thought to restore gastric retention and thus prevent the rapid passage into the small bowel. Techniques used for TORe include argon plasma coagulation (APC) and/or endoscopic suturing. Retrospective studies have shown promising results in the reduction of the anastomotic diameter as well as on DS and PBH. 3 4 5 6 7 8 In 2010, Heylen showed that TORe with a conventional over-the-scope clip is technically feasible and results in a positive effect on weight regain. 9 These results led to the development of the Bariatric Reduction System, which is a dedicated device for clip-based TORe. Up to now, only one small preclinical study and a small case series have been performed and they showed promising results on reduction of the anastomotic diameter. 10 11 Here, we report the results of a prospective multicenter study to investigate the technical feasibility and a possible clinical impact of clip-based TORe in patients with DS and PBH.

## Materials and Methods

### Study Design

This was a prospective, single-arm clinical trial, including patients suffering from DS and/or PBH after RYGB at three referral centers in Germany. The study was approved by the Institutional Review Board (No. 443/19; May 12, 2020). All patients gave written consent after the explanation of possible treatment alternatives. Patients had the right to withdraw consent at any time.

In all, 20 subjects were planned, and a 90% success rate was assumed. To estimate with reasonable precision, the Wilson confidence interval was chosen, which was the following: 0.6990–0.9721.

The study population was extended to *n* = 23 as by the time the number of *n* = 20 was reached, three other patients were already screened, and the decision was made to include those patients as well. A formal power calculation was not performed.

### Inclusion/Exclusion Criteria

Adult patients with evidence of DS and/or PBH and previous unsuccessful dietary or medical treatment were included in the study. Criteria for DS and/or PBH were: combination of Sigstad Score ≥7 and a pathological result in the modified oral glucose tolerance test (mod oGTT). For DS, a pathologic result was defined as increase in heart rate by 10% and/or a drop in systolic blood pressure by 10% within the first 30 min. For PBH, a serum glucose value <60 mg/dL/ (<3.3 mmol/L) was diagnostic as described elsewhere or if patients had severe symptoms of hypoglycemia. 1 Exclusion criteria were known esophageal stenosis, varices, inability to give informed consent, and an insulin-dependent diabetes mellitus. Prior surgical revision and/or pouch dilation were not exclusion criteria.

### Endpoints and Definitions

The primary endpoint of the study was short-term feasibility. This was defined as reduction of the anastomotic diameter below 15 mm and/or reduction of the anastomotic surface area of at least 70%. Measurement was performed by applying resection snares or an opened forceps with defined diameter. In detail, e.g., a 15 mm snare would be opened and held onto the ring of the anastomosis. If the anastomosis would be larger, the distance was estimated.

Secondary endpoints included procedural data and adverse events. Time of the esophagogastroduodenoscopy (EGD) was counted from first insertion of the endoscope until final withdrawal. Time of TORe was defined from reinsertion of the endoscope equipped with the system until clip deployment. Any adverse events during or within the follow-up period were recorded. Severe adverse events were defined as any event being life-threatening, prolonging hospital stay, requiring readmission to the hospital, or leading to permanent impairment or death.

**Fig. 1:**
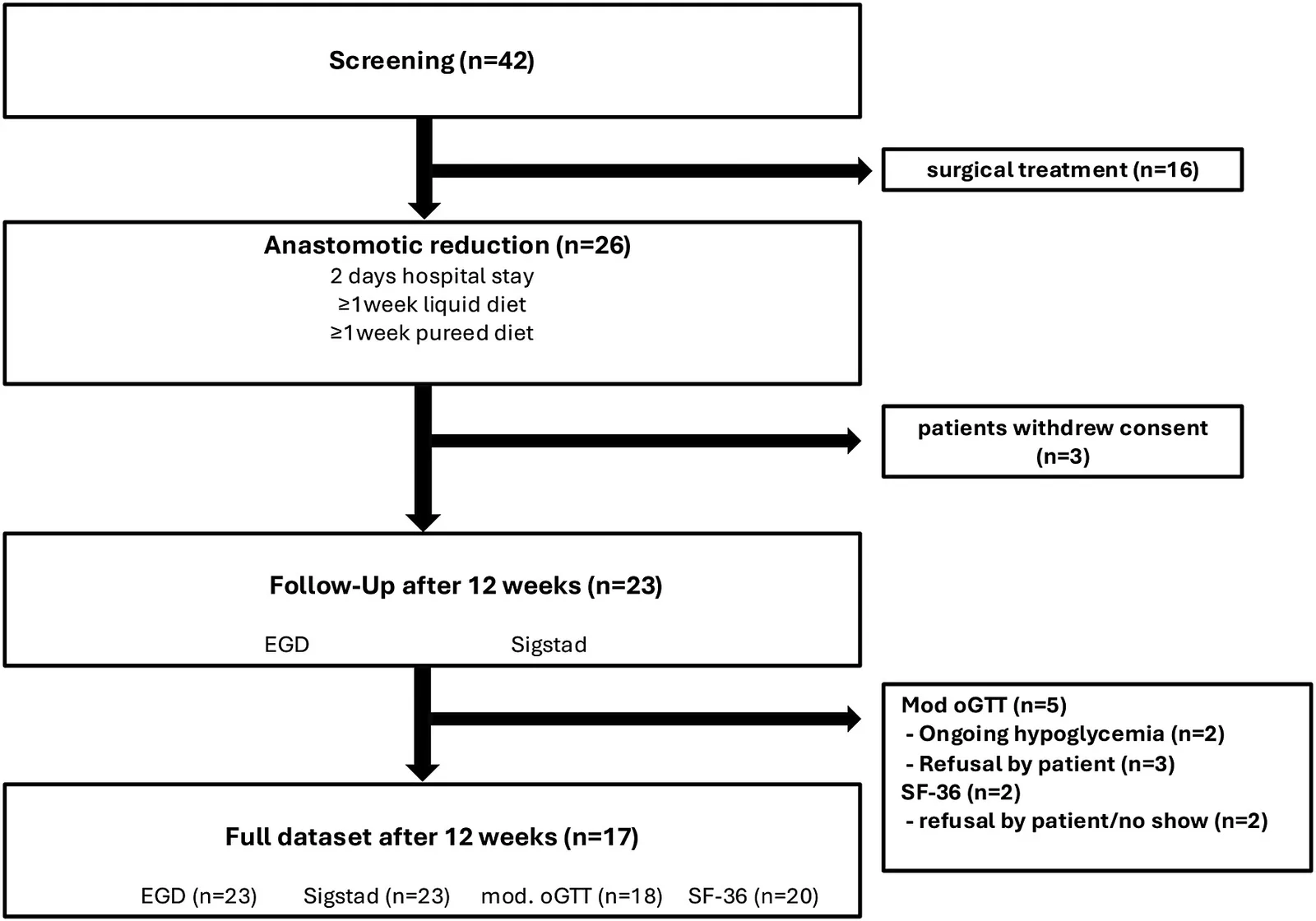
A flowchart of the study. In all, 42 patients were screened for eligibility. In *n* = 26, an anastomotic reduction was performed. Three patients withdrew their consent, resulting in *n* = 23 that were available for analysis. Five patients did not receive mod oGTT, two of them due to ongoing hypoglycemia during follow-up. In these cases, the mod.oGTT was not attempted after 12 weeks. Three patients refused to do the test. EGD = esophagogastroduodenoscopy; Sigstad = Sigstad Score; mod oGTT = modified oral glucose tolerance test; SF-36 = Short-form 36 (Quality of life).

Patients underwent clinical and endoscopic follow-up at 12 weeks after the procedure. Change in anastomotic diameter, symptom scores, mod. oGTT, BMI, and quality of life (QoL; SF-36) were assessed ( Fig. 1 ).

### Procedure and Postoperative Course

**Fig. 2:**
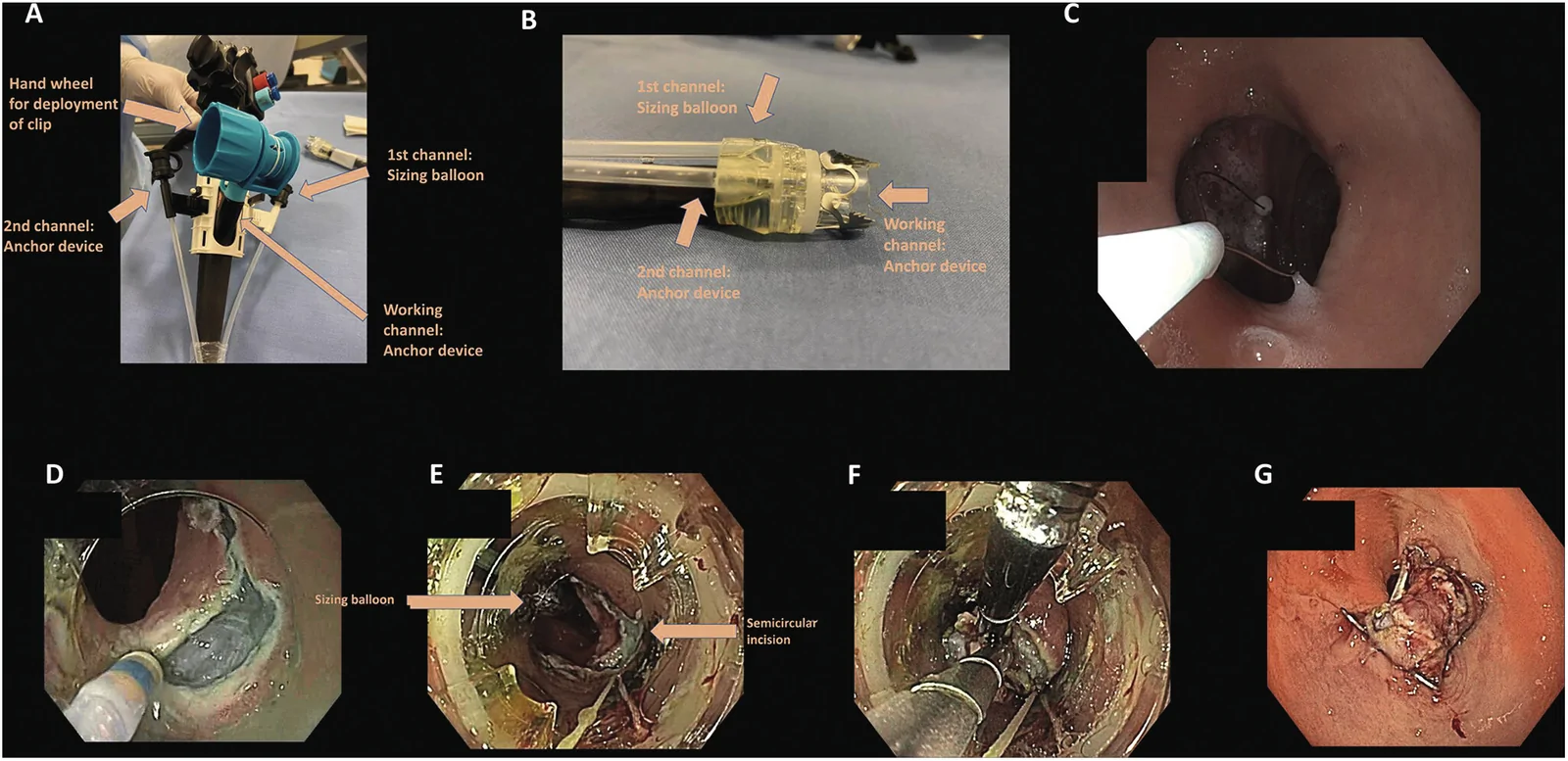
Assembly of the system ( **A** + **B** ) and application of clip ( **C** – **G** ). **A** A handwheel is mounted on endoscope working channel. Additional channels for sizing balloon and anchor are attached. **B** Distal end with cap and different channels. **C** Measuring the diameter of the anastomosis with snare. **D** Semicircular incision with ESD knife. **E** Mounted system with cap. Sizing balloon is at the end of the cap and semicircular incision point is indicating anastomosis. **F** Anchors placed crossover and tissue is mobilized. **G** Clip deployed.

Bariatric reduction system consists of a modified over-the-scope clip with additional teeth to ensure firm tissue grabbing (see also Fig. 2 and supplemental Video 1). The cap is longer and larger in diameter. The tissue is mobilized with two anchors – one of them running through the working channel of the endoscope, the other one running externally on the endoscope in an additional working channel that ends in the transparent cap. A third channel runs externally on the endoscope. This channel is used for a wire-guided “spacekeeper” balloon to prevent complete closure of the anastomosis.

After measuring the diameter, the anastomosis is prepared by mucosal incision: after injection of saline and methylene blue, a semicircumferential incision (or two separate spots of 5 mm length) e.g., with an ESD knife (Dual Knife; *ERBE Vio3 generator and EndoCut Q* [ *Effect 2, Duration 3, Interval 3* ]) at 3–5 mm distance to the anastomotic ring is performed to expose the submucosa. The endoscope is then withdrawn and the device is loaded. Reinsertion and passage of the upper esophageal sphincter (UES) can be managed by gentle pushing or with a 20 mm balloon. The next step is placing the spacekeeper balloon over the anastomosis. Anchors are then pushed into the preconditioned spots: as the anchors should be placed on opposite sides of the anastomosis to ensure maneuverability, the lower spot is where the working channel ends and the upper spot where the additional channel ends (e.g., 7 o’clock and 1 o’clock position for Olympus Endoscopes). The anchors are then gently pulled into the cap, thereby narrowing the two sides of the anastomosis until the cap is either fully loaded with tissue or at least the anchors are seen on the edge of the cap to prevent clip capture of the anchors. The clip is released by turning the hand wheel clockwise. The 6 mm spacekeeper-balloon (inflated to 6 atm) secures a residual lumen of the anastomosis.

After the intervention, patients stayed in the hospital for two days for conservative treatment, and a second look EGD was performed after 48 h if clinically indicated. After discharge, the patients stayed on a liquid diet for at least one week followed by a soft diet for another week.

### Statistical Analysis

Categoric variables are expressed as frequencies and percentages, and metric variables are expressed as median with interquartile range. Changes of study parameters pre-bariatric reduction system (BARS), post-BARS, and 12 weeks after the intervention were analyzed using the Wilcoxon signed-rank test or Friedman test as applicable. Statistical analyses were performed using SPSS (version 28.0, IBM, New York, US) and GraphPad Prism (version 8, GraphPad Software, San Diego, US).

## Results

### Study Enrolment and Patient Characteristics

Between September 2020 and January 2024, 23 patients were enrolled at three study centers in Germany. Baseline patient characteristics and the flowchart are shown in Table 1 and Fig. 1 .

**Table 1:** Baseline patient characteristics.

Baseline patient characteristics ( *n* = 23)			
Age {years; median + IQR}		39.0	35–49
Sex {female vs. male, %}		19 (83%)	4 (17%)
BMI at baseline {kg/m ^2^ ; median + IQR}		30.6	28–35.8
Art. HTN		8	35%
Coronary artery disease { *n* ;%}		0	0%
T2DM		3	13%
Steatosis hepatis { *n* ;%}		5	22%
Obstructive sleep apnea { *n* ;%}		0	0%
Depression { *n* ;%}		2	9%
Refluxesophagitis { *n* ;%}		1	4%
Any comorbidity { *n* ;%}		11	48%
Onset symptoms after RYGB {days; median + IQR}		981	534–1826
Treatment of DS and PBH			
	Dietary { *n* ;%}	23	100%
	Acarbose { *n* ;%}	18	78%
Sigstad Score {median + IQR}		17.5	12.8–21.0
Mod oGTT			
	DS { *n* ;%}	22	96%
	PBH { *n* ;%}	19	82%
	Combined { *n* ;%}	18	78%

IQR = Interquartile Range; BMI = Body Mass Index; HTN = Arterial Hypertension; T2DM = Type 2 Diabetes Mellitus; RYGB = Roux-en-Y Gastric Bypass; DS = Dumping Syndrome; PBH = Post Bariatric Hypoglycemia; mod. oGTT = Modified oral Glucose Tolerance Test.

Modified oral glucose tolerance test was performed as described in the “Methods” section.

### Primary Endpoint and Procedural Data

The clip could be deployed in all cases (100%). After the intervention, the median anastomotic diameter decreased significantly from 40 mm (IQR: 30–50) to 9 mm (IQR: 7–10), and anastomotic surface from 1243.8 mm ^2^ (IQR: 706.9–1963.5) to 63.6 mm ^2^ (IQR: 38.5–78.5) ( *p* < 0.001), resulting in a relative reduction of the surface area of 93.2% ± 0.9% (95% CI: 91.1–95.2%). Results are also shown in Table 2 .

**Table 2:** Primary endpoint and procedural data.

Primary endpoint and procedural data					
Anastomotic diameter {mm; median + IQR}		Pre	40	30–50	
		Post	9	7–10	*p* < 0.001
Anastomotic surface {mm ^2^ ; median + IQR}		Pre	1243.8	706.9–1963.5	
		Post	63.6	38.5–78.5	*p* < 0.001
Preparation of Anastomosis					
	Incision of Anastomosis { *n* ;%}		23	100%	
		Semicircumferential { *n* ;%}	8	35%	
		Two separate spots { *n* ;%}	15	65%	
Passage of the UES					
	Insertion balloon needed {n;%}		16	70%	
	No extra device {n;%}		7	30%	
Anchors					
	Anchors straight {n;%}		3	13%	
	Anchors crossed {n;%}		20	87%	
Clip deployment successful { *n* ;%}			23	100%	
Time EGD {minutes; median + IQR}			37	29–55	
Time BARS {minutes; median + IQR}			8	5–19	
Sedation					
	Propofol only { *n* ;%}		14	61%	
	Midazolam only { *n* ;%}		2	9%	
	Propo + Mida { *n* ;%}		4	17%	
	General Anesthesia { *n* ;%}		3	13%	
Technical Difficulties					
	Spacekeeper balloon not inflating { *n* ;%}		2	9%	
Complications					
	Subtotal closure of anastomosis { *n* ;%}		1	4%	

Table 2 : Primary endpoint and procedural data. Primary endpoint was defined as reduction of the anastomotic diameter <15 mm or ≥70% reduction of surface area. UES = upper esophageal sphincter; SD = Standard deviation; EGD = esophagogastroduodenoscopy; BARS = Bariatric Reduction System; *p* -value ≤ 0.05 significant.

Median whole procedure time was 37 min (IQR: 29–55) and the median time for the clip procedure was 8 min (IQR: 5–19). In 20 of the 23 patients (87%), the procedure was performed under conscious sedation, while in 3 of the 23 patients (13%), the procedure was performed under general anesthesia. The anastomosis was pretreated by mucosal incision in all cases. The UES was passed by using the insertion balloon in 16 of the 23 patients (70%), while in the rest of the cases, no extra device was needed. Clip deployment was successful in all 23 cases (100%). In 2 of the 23 patients, there was a malfunction of the spacekeeper balloon, which did not inflate (9%). In one of these cases, an almost complete closure of the anastomosis occurred (4%).

### Secondary Endpoints

**Fig. 3:**
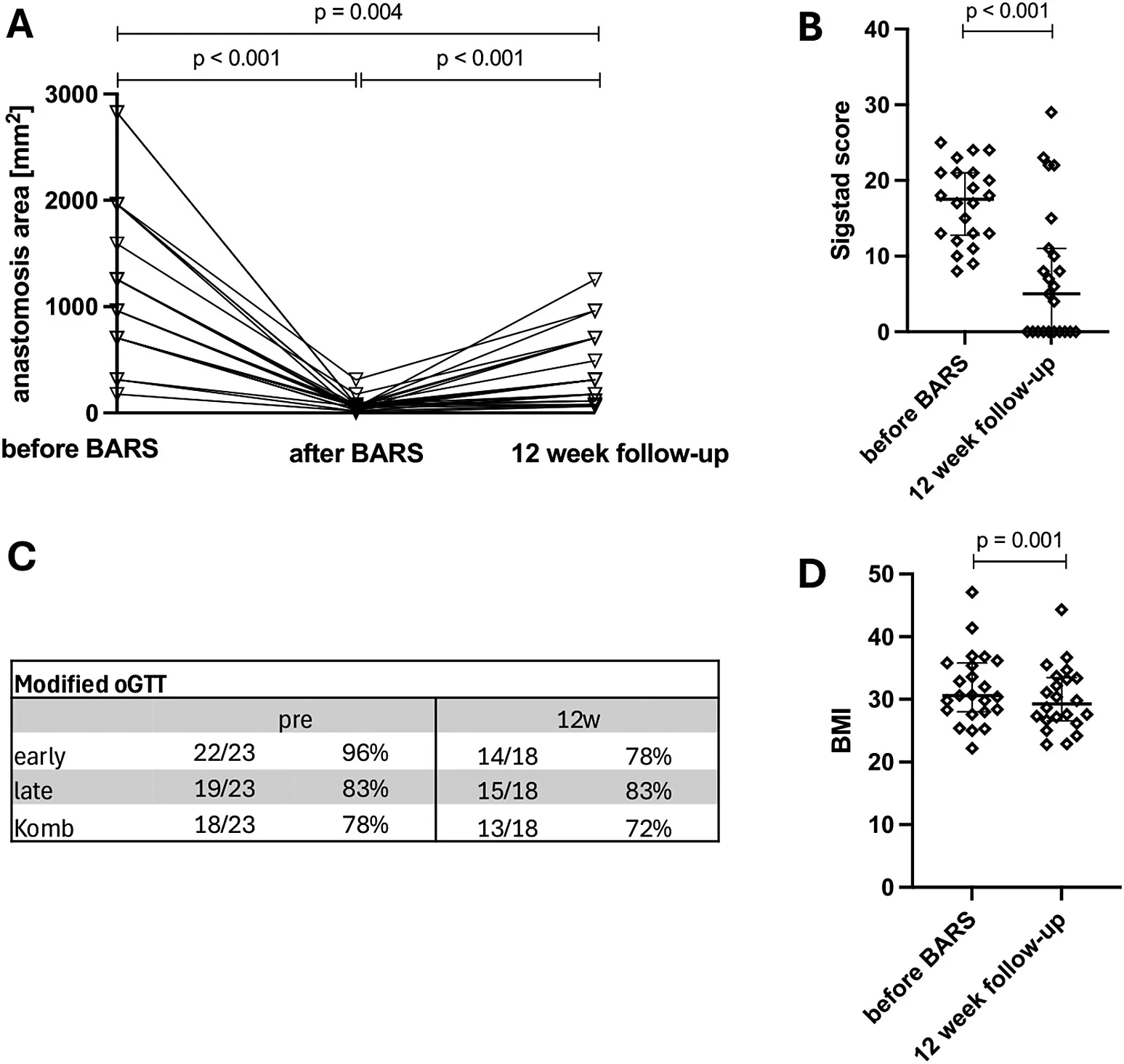
Clinical outcome. ( **A** ) Anastomotic surface before and after BARS and at 12 week follow-up. ( **B** ) Sigstad Scores before BARS and at 12w follow-up. **C** Results of the modified oGTT. Absolute numbers in relation to the patients and their percentage are shown. ( **D** ) BMI before and after 12 week follow-up.

After 12 weeks, the median anastomotic diameter was found to be 20 mm (IQR: 12–30 mm) and anastomotic surface was 314.2 mm ^2^ (IQR: 113–707). Compared to median baseline (diameter 40 mm (IQR: 30–50); surface 1243.8 mm ^2^ [IQR: 706.9–1963.5]), there was a significant reduction of both parameters ( *p* < 0.001), resulting in a relative reduction of surface area of 59.6% ± 6.8% (45.5–73.75%). In contrast to the immediate reduction in surface and anastomotic diameter, there was a significant increase at week 12. As shown in Fig. 3 and **Supplementary Table 1** , the anastomotic diameter was as high as the baseline and the clip was detached in four patients (17%).

### Sigstad Score

Median Sigstad-Score at baseline was 17.5 (IQR: 13–21) and decreased to 5 (IQR: 0–11) at week 12. This difference was statistically significant ( *p* < 0.001). The mean relative reduction was 59% ± 9% (95% CI 39–79%).

### Modified oral Glucose Tolerance Test

In the modified oGTT, DS dumping occurred in 14 out of 18 patients at week 12 (78%), leading to a reduction of 18% compared to baseline. Regarding PBH, the rate of patients with a pathologic result was unchanged at follow-up. Out of the 18 of 23 patients with a combination of DS and PBH (78%), at baseline, 13 of 18 (72%) still had combined disease as one patient improved his DS.

### Quality of Life

There was an increase in all subcategories of SF36 ranging from 13% to 100% except “emotional role function,” which had a median maximum reachable value of 100 points already at baseline ( Table 3 ). The difference was statistically significant in “role function” (100% increase, *p* = 0.05), “bodily pain” (45%; *p* = 0.002), “vitality” (57%; *p* = 0.016), “social function” (40%; *p* = 0.016), and “mental health” (13%; *p* = 0.028).

**Table 3:** Quality-of-life scoring (SF 36).

Subcategory		Baseline	12 Weeks	Relative increase	*p* -Value
Physical function	(Median; IQR)	75 (65–90)	90 (80–95)	20%	0.099
Role Function	(Median; IQR)	50 (0–100)	100 (50–100)	100%	**0.05**
Bodily Pain	(Median; IQR)	51 (31–74)	74 (51–100)	45%	**0.002**
General Health	(Median; IQR)	52 (45–72)	62 (52–72)	19%	0.146
Vitality	(Median; IQR)	35 (20–50)	55 (30–65)	57%	**0.016**
Social function	(Median; IQR)	63 (25–88)	88 (50–88)	40%	**0.016**
Emotional role function	(Median; IQR)	100 (67–100)	100 (100–100)	0%	0.143
Mental Health	(Median; IQR)	64 (48–72)	72 (56–84)	13%	**0.028**

Table 3 : Results of the Quality of Life Scoring from Baseline to 12w Follow-up. SF-36 = Short-Form 36; IQR = interquartile range. *p* -Value ≤ 0.05 significant.

### BMI

The median BMI of the patients decreased from 30.60 kg/m ^2^ (IQR: 28.00–35.80) to 29.25 kg/m ^2^ (IQR: 26.70–33.40) within 12 weeks ( *p* = 0.001). The mean relative reduction was 6% ± 1.3% (95% CI 3.6–9.2%).

### Adverse Events

Within the first 24 h post intervention, 18 of the 23 patients (78%) suffered from epigastric pain, gradually decreasing from day 2 (6 of 23; 26%) to week 12 (3 of 23; 13%). Baseline pain scores were not evaluated. Nausea was present in 13 of 23 patients (57%) immediately after the procedure and decreased rapidly thereafter with 1 of 23 patients at day 2 (4%) and 2 of 23 patients at week 12 (9%) still complaining about nausea. A single patient suffered from vomiting after the intervention, which ceased on day 2 but reoccurred at follow-up, however, with no relation to the anastomosis.

In 2 of the 23 patients, severe adverse events were reported (9%), while one of them was not related to the intervention (worsening diabetic foot). The other patient was readmitted to the hospital 4 weeks after the intervention with anemia. A potential cause could have been an anastomotic ulcer while no signs of bleeding were seen in EGD (see also **Supplementary Table 2** ).

## Discussion

**Video 1** Clip-based anastomotic reduction is shown. Essential steps are: measurement of the diameter, semicircular mucosal incision, assembly of the system, anchor placement, clip application with spacekeeper balloon in place, reinspection.



This is the first prospective study on the feasibility of clip-based TORe in the context of DS and PBH. Our results indicate that reduction of the anastomotic diameter with the clip-based system is technically feasible and seems safe in the short-term follow-up. Moreover, Sigstad symptom score and subcategories of QoL scores improved significantly.

With respect to the baseline characteristics, our study cohort is a little younger and has a modest median BMI and fewer comorbidities compared to previous publications on TORe with endoscopic suturing in the context of DS. 3 4 5 6 7 8

The primary endpoint of the study was reached in all cases. The optimal postinterventional diameter of the anastomosis to effectively delay gastric emptying is not defined for DS and PBH. Previous studies indicate that a reduction below 15 mm may be sufficient for treatment of weight regain, which could function as a surrogate for DS and PBH. 12 On the other hand, in case of a very large anastomosis (up to 60 mm in our study), a reduction to ≤15 mm is sometimes not applicable with a single intervention. Therefore, we assumed that a reduction ≥70% of the anastomosis surface is sufficient and chose this second criterion.

The reduction in anastomotic diameter surpassed the results published in a preclinical study on TORe with the BARS 11 and was comparable to the larger retrospective case series of patients treated with a conventional over-the-scope-clip. 9 Of note, both studies were performed in the setting weight regain after RYGB and one was mainly performed on porcine models. 11

In contrast to these two studies, general anesthesia was not necessary in most cases in our cohort. Although general anesthesia can be helpful for performing the intervention, staff resources and costs are higher compared to propofol sedation. Therefore, it may be restricted to patients with high risk for sedation.

We conditioned the anastomosis with mucosal incision to ensure better fixation of the anchors. This concept has been shown to be effective in other TORe studies and was routinely done in all cases. 13 14 In our opinion, the clip-based method seems simpler and may be easier to learn compared to suture-based TORe, but an existing set of skills is mandatory such as mucosal incision, anchor placement, and clip application. Although not systematically evaluated in this study, a guess would be that 3–5 interventions are needed to feel safe, which would be a lot faster than with suture-based TORe. 15 However, as the broad spectrum of hospitals does not provide such skills, e.g., over-the-scope-clip application, the technique is currently limited to specialized centers.

Data from our study indicate that the system is well tolerated, given the low rates of adverse events. There were two cases in which the spacekeeper balloon did not inflate properly, resulting in near closure of the anastomosis in one case. This case was managed conservatively without additional intervention. Postinterventional complaints such as nausea (13 of 23 patients; 57%) and pain (18 of 23 patients; 78%) were frequently observed during the first 24 h but gradually decreased over time and could be managed conservatively.

At 12 week follow-up, the anastomotic diameter was still reduced significantly compared to the baseline.

**Fig. 4:**
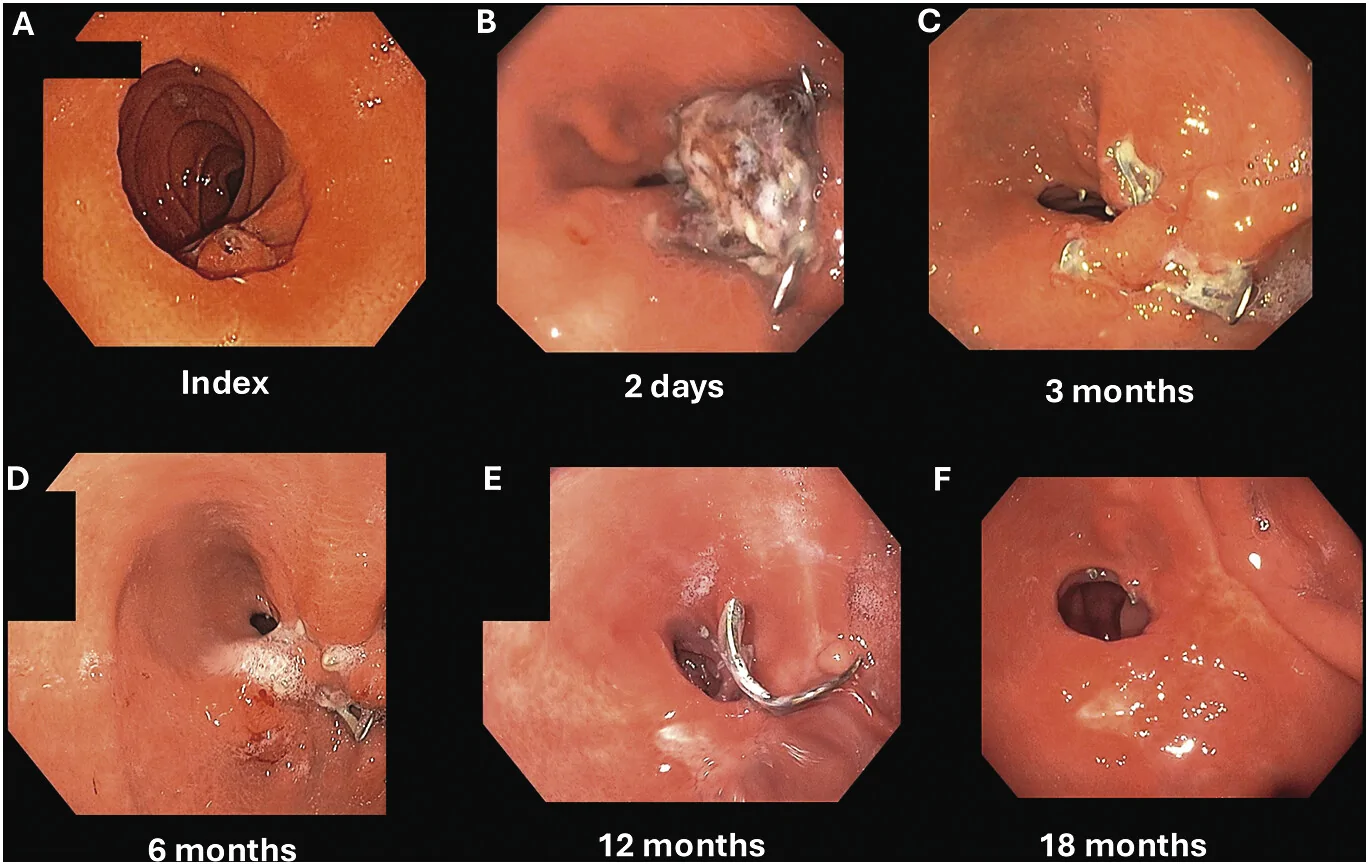
Long-term follow-up of anastomotic diameter and clip. The anastomotic area is shown at Index ( **A** ), after two days ( **B** ), 3 months ( **C** ), 6 months ( **D** ), 12 months ( **E** ), and 18 months ( **F** ). While there is stable reduction in anastomotic diameter, the clip gradually moves into the lumen and detaches. Images **D** – **E** – **F** are beyond the study protocol and are meant to illustrate the process of clip loss while still maintaining a narrowed anastomosis.

However, there was a significant enlargement compared to the immediate reduction of the anastomosis. It has to be noted that the diameter immediately after application of the clip is also influenced by edema related to preconditioning as well as manipulation of the tissue with anchors. It is therefore not surprising that it naturally enlarges over time (see also Fig. 4A–C ). As our data show, the median diameter was still significantly reduced by 50% after 12 weeks. A definite diameter that is necessary for a clinical effect in DS and PBH has not been described yet. As none of the other studies on clip-based TORe have done a structured endoscopic workup of the anastomotic reduction, a comparison cannot be made. 9 10 11 However, the enlargement over time harbors the risk of loss of effect. We cannot exclude a deterioration of symptoms between clip placement and after 12 weeks. Moreover, long-term efficacy was not tested in this study. We believe that the reduction of the diameter together with the results of the Sigstad score, the decline in BMI, and improvement in QoL could indicate a clinical short-term effect.

Surprisingly, the clip was not detectable in 11 of the 23 patients (46%) at follow-up but the anastomotic diameter was still reduced in all but 4 of the 23 cases (18%). An explanation could be that the detachment of the clips in the four cases happened early after the procedure as a “tear off” while in the other 6 of the 23 patients (26%), the clip gradually moved into the lumen leaving a narrowed anastomosis (see also in Fig. 4D–F showing a longer follow-up beyond the study period as an illustrative case; see also Video 1 ). Hence, clip detachment per se does not necessarily mean treatment failure; it is actually a common phenomenon after over-the-scope clip placement. 16 17 It also matches with the study by van Heylen who could only detect 29% of the clips on X-ray after three months despite the fact that the clinical effect of weight loss lasted over 12 months. However, a 18% tear-off rate is fairly high, limiting the durability of the method. Due to the small sample size of our study, predictors of failure could not be reliably calculated.

Our study is the first to evaluate the clinical effect of clip-based anastomotic reduction on DS and PBH. Several studies have reported results of suture-based TORe for this indication. 3 4 5 6 7 8 18 The size of study cohorts ranged from 11 7 to 115 patients 4 with follow-ups between 1 month 3 4 up to 3.45 years. 18 All studies showed an improvement on DS and in those where the Sigstad score was calculated, the improvement at follow-up was statistically significant and similar to our results. In the remaining three studies, only an improvement in symptoms at follow-up is stated but no calculation of any score has been performed. 7 8 18

Surprisingly, oGTT results did not correlate with improvements in Sigstad scores and anastomotic reduction. The measured effect on DS was moderate and for PBH, no change in oGTT was seen at all. This questions the objective effect of the therapy and a possible influence of a placebo effect. Due to the lack of a (sham-) control group, we can neither exclude this possibility nor the fact that symptom scores exaggerate a minor effect. On the other hand, mod oGTT is suitable for diagnosis in combination with symptoms, and changes in mod oGTT are shown in pharmacologic studies. 19 20 21 In the context of TORe, the test has not been performed yet. It is therefore unclear if it is suitable to objectively investigate clinical effectiveness of TORe. A recently published study on pouch resizing in patients with DS and PBH with a similar workup also found no change in glycemic control or heart rate 12 months after the procedure despite relevant improvement of symptom scores. 22

A possible explanation could be that patients with a regain of storage gastric function just eat smaller meals, and the fluid consistency of the test meal passes the anastomosis too quick. In both studies, diet was not systematically recorded. In favor of this theory is the reduction in symptom scores together with the documented weight loss. Future studies should switch to food that is closer to a solid food as well as to use continuous glucose monitoring for detection of changes in glycemic control.

Dumping syndrome after bariatric surgery can significantly reduce QoL. 23 Our study demonstrated an increase in nearly all subcategories of the SF36. Compared to a retrospective study comparing SF36 values in the context of dumping after sleeve gastrectomy, the significant improvements align with the subcategories mental health and vitality. Although the SF36 is not specifically designed for DS and therefore not all subcategories are directly applicable, vitality and role function (showing improvement of +100% and +57%, respectively) appear particularly suited to reflect the effects of TORe, as they include questions related to concentration and the ability to perform daily activities. However, it has to be noted that a dumping-specific minimal clinically important difference (MCID) is not described in the literature. A systematic review in another clinical context found a roughly five-point increase corresponds to an MCID, which is met by the described categories. 24

As a “bystander effect”, our study showed a median reduction in BMI at 3 months of 1.35 kg/m ^2^ ( *p* < 0.001). This matches with the above-mentioned studies on TORe in the context of dumping and might be an (objective) indicator (besides symptoms) that restrictive function has been restored.

A cost-effectiveness analysis of BARS to treatment alternatives was not part of this study. Although long-term data are absent, a potential benefit of the system compared to suture-based TORe is the possible lower cost of the procedure itself as reimbursement – at least in Germany – is beneficial.

Our study has several limitations.

- Small sample size: the focus of the study was technical feasibility, for which the sample size was calculated. As a consequence, secondary endpoints regarding clinical efficacy may be underpowered. Furthermore, subgroup analyses (e.g., DS-only vs. PBH-only vs. both) or predictors of failure/success were not amendable to calculate. In addition, multiple comparisons (e.g., SF-36 subscales) inherit the risk of false positive findings and therefore have to be interpreted with caution.
- Single-arm design: with the absence of a control group *,* key confounders (e.g., placebo effect) are not controlled for, questioning the generalizability of the results on clinical outcome.
- Missing data: a complete data set for all intended analyses was not available as some patients were not fully adherent to the study protocol, increasing the risk of bias in interpreting the effect. In detail, five patients did not receive a mod. oGTT after 12 weeks. In order to assess the effect of missing data, a best-case/worst case scenario was calculated. Regarding DS, missing data could have a range of influence of 22% in DS, 18% for PBH, and 22% for combined disease. When these five patients are compared to the rest of the cohort, their anastomoses were smaller, but their relative reduction after 12 weeks were comparable. Median Sigstad score reduction was smaller (18 → 11). Moreover, 2 of these 5 patients had an anastomotic enlargement back to baseline after 12 weeks. They also had no improvement on the Sigstad Score and did not want to fulfill SF-36 as well as oGTT testing. Another two patients that did not undergo oGTT had ongoing symptoms of PBH and therefore mod. oGTT was not attempted (one of them with no change in Sigstad). The last participant who did not show up for testing after 12 weeks had a stable anastomotic reduction (30 mm → 10 mm → 12 mm), as well as an improvement in the Dumping Scale (15 → 4) but simply did not want to go to the test. In summary, this patient group had worse clinical outcomes compared to the rest of the cohort, and therefore a complete dataset would have been desirable. Their parameters such as anastomotic diameter and Sigstad Score went into the analysis of the whole cohort to minimize a bias.
- Short follow-up: long-term durability, cost-effectiveness, long-term adverse events, or the need for repeat procedures could not be shown. A long-term analysis including all indications for TORe is planned to address this issue.
- Measurement: anastomotic diameter by snares/forceps lacks accuracy and has a possible interobserver variability. As re-measurements were taken by the same endoscopists at their sites, the interpatient variability might not be influenced as much.
- Manufacturer-linked bias: as in every industry-funded study with disclosed author financial relationships, there is a risk of selection-, measurement-, or reporting bias. This stresses the need for independent further investigation of the device.
- Adverse event-reporting: while SAEs had a clear definition, for AE, there was no such definition (e.g., Clavien-Dindo), resulting in loss of standardization of reporting.
- Generalizability: with the limitations mentioned above taken into consideration along with the fact that a special skillset is needed as a basis for the technique, the results found in the study might not be generalizable and may not be applicable to other health care systems.

In conclusion, this first prospective study on clip-based TORe demonstrates technical feasibility and tolerability of the device in the short-term follow-up. Within a 3-month FU, anastomotic reduction was observed and could be associated with improvement in symptom scores and QoL. Larger studies with longer follow-ups are needed to further evaluate the clinical effect of this promising approach before randomized sham-controlled trials (e.g., clip-based vs. sham or clip vs. suture-based TORe) need to be conducted.

## Supplementary Material

Ergänzendes Material Supplementary Material
