# Supplementary material for: Clip-Based Endoscopic Anastomotic Reduction in Patients with Dumping Syndrome and Postbariatric Hypoglycemia after Roux-en-Y Gastric Bypass
Source: Endosc Int Open. 2026 Aug 20;14:a29317366. doi: 10.1055/a-2931-7366 (PMC13552001; doi:10.1055/a-2931-7366)
Supplement: Supplementary file 1 [file 10-1055-a-2931-7366_29336511.pdf]

Clip-based Endoscopic Anastomotic Reduction in Patients with Dumping Syndrome and Post-bariatric Hypoglycemia after Roux-en-Y Gastric Bypass

Supplementary Table 1:
Outcome

Outcome											
Parameter	Baseline			Post			12w			Base -> 12w	Post ->12w
	Median	25th	75th	Median	25th	75th	Median	25th	75th		
Radius {mm}	20.00	15.00	25.00	4.50	3.50	5.00	10.00	6.00	15.00	$p < 0.001$	$p < 0.001$
Surface {mm2}	1243.8	706.90	1963.50	63.60	38.50	78.50	314.20	113.10	706.90	$p < 0.001$	$p < 0.001$
Sigstad	17.50	13.00	21.00				5.00	0.00	11.00	$p < 0.001$	
BMI {kg|m2}	30.60	28.00	35.80				29.25	26.70	33.40	$p = 0.001$	

Supplementary Table 2:
Adverse events

Adverse events						
	Day 1		Day 2		12W Follow-Up	
Pain (n=patients; %)	18	78%	6	26%	3	13%
Nausea (n=patients; %)	13	57%	1	4%	2	9%
Vomiting (n=patients; %)	1	4%	0	0%	1	4%
Severe adverse events						
Anemia	1	4%	possibly related with Ulcer from ESD/Clips			
Other	1	4%	unrelated with worsening diabetic foot syndrome			
